# Prevalence and Clinical Features of Portopulmonary Hypertension in Patients With Hepatic Cirrhosis: An Echocardiographic Study

**DOI:** 10.7759/cureus.24957

**Published:** 2022-05-13

**Authors:** Anany Gupta, Akshyaya Pradhan, Sanjay Mehrotra, Ravi Misra, Kauser Usman, Ajay Kumar, Shivani Pandey

**Affiliations:** 1 Medicine, King George's Medical University, Lucknow, IND; 2 Cardiology, King George's Medical University, Lucknow, IND; 3 Biochemistry, King George's Medical University, Lucknow, IND

**Keywords:** pulmonary vascular resistance, pulmonary wedge pressure, pulmonary hypertension, portal hypertension, liver cirrhosis

## Abstract

Objective

The present study was conducted to delineate the prevalence and clinical features of portopulmonary hypertension in patients with hepatic cirrhosis. Possible associations between echocardiographic variables and portopulmonary hypertension were also explored.

Methods

A prospective, observational study was conducted between September 2017 and August 2018. Differences in demographics, clinical presentation, laboratory findings, and echocardiographic findings in cirrhosis patients with and without portopulmonary hypertension were compared.

Results

The prevalence of portopulmonary hypertension in patients with hepatic cirrhosis was found to be 9.3%. Hemoglobin was significantly lower among patients with portopulmonary hypertension compared to those without portopulmonary hypertension (5.50±0.68 g/dl vs. 7.26±1.43 g/dl, p=0.001). All patients with portopulmonary hypertension displayed right atrial (major: p=0.0001 and minor: p=0.001) and right ventricular (basal, p=0.0001; longitudinal, p=0.0001) dilation. Several variables such as right ventricular systolic pressure (p=0.0001), pulmonary artery diameter (major: p=0.0001; right: p=0.0001; and left: p=0.007), pulmonary vascular resistance (p=0.0001), tricuspid regurgitation (p=0.0001), pulmonary regurgitation peak pressure gradient (p=0.0001), pulmonary regurgitation end diastolic gradient (p=0.0001), left atrial dimension (major axis: p=0.002), left atrial volume (p=0.04), left ventricular outflow tract (p=0.001), inferior vena cava diameter (p=0.001), and inferior vena cava collapsibility (p=0.001) were higher in patients with portopulmonary hypertension compared to patients without portopulmonary hypertension.

Conclusions

The present study revealed a 9.3% prevalence of portopulmonary hypertension among patients with hepatic cirrhosis. Patients with portopulmonary hypertension displayed significantly lower haemoglobin levels, right and left ventricular dilation, and higher values of several echocardiographic variables as compared to those without portopulmonary hypertension.

## Introduction

Portopulmonary hypertension occurs due to coexistence of pulmonary hypertension and portal hypertension. Despite prevalence in patients with and without hepatic cirrhosis, it is more often observed in the former [1]. Etiologies of hepatic cirrhosis include excessive alcohol intake, viral infections (hepatitis B, hepatitis C, or their combination), autoimmune diseases, and cryptogenic causes [2]. Dyspnea is the most frequent documented symptom however, in some asymptomatic cases, right heart function may decline with gradual increase in pulmonary vascular resistance [3]. Echocardiography has been the preferred diagnostic modality employed to screen patients; however, right heart catheterization is the gold standard [2]. Portopulmonary hypertension is hemodynamically defined as increased mean pulmonary arterial pressure ≥25 mmHg, pulmonary arterial wedge pressure >15 mmHg, and pulmonary vascular resistance 3 Wood units (WU) [4]. There are only two treatment strategies, these are targeted therapy of pulmonary hypertension or liver transplantation [5]. However, 14% survival rate for untreated portopulmonary hypertension, 45% survival rate for only pulmonary artery pressure treatment, and 67% survival rate for liver transplantation reveal a dismal prognosis for these patients [6].

Portopulmonary hypertension remains a vastly unexplored condition, especially in the Indian population. The low incidence of this condition eludes its prevalence as well as clear understanding of its clinical features which are yet to be delineated in their entirety. Moreover, the scarcely available clinical data are inconsistent in terms of study design, sample size, patient population, clinical features, protocol/guideline adherence, and diagnostic techniques. All these inconsistencies provide a rather vague depiction of this condition. Against this background the present study was conducted to (i) determine the prevalence of portopulmonary hypertension in patients with hepatic cirrhosis and (ii) determine the association of portopulmonary hypertension in cirrhotic patients, with respect to clinical features and echocardiographic variables.

## Materials and methods

Study design and patient population

This prospective, observational study was conducted at a tertiary-care centre in India during the study duration between September 2017 and August 2018. King George's Medical University Institutional Ethics Committee issued approval ECR/262/Inst/UP/2013/RR-16. All patients provided written informed consent for study participation. Patients from the Inpatient Department aged 18 to 65 years with chronic liver disorders (eg. jaundice, edema, ascites, hypersplenism, lower esophageal varices, rectal varices, deranged liver function tests and Child-Pugh scores, surface nodularity and coarse and heterogeneous liver texture determined by ultrasonography) were included in this study.

The exclusion criteria included patients with congenital heart disease, valvular heart disease, pulmonary veno-occlusive disease, left ventricular disease, pulmonary capillary hemangiomatosis, lung disease, lung and liver transplantation, liver tumours, spleen resection, connective tissue disorders, thyroid gland disorders, sickle-cell disease and related conditions, sarcoidosis, human immunodeficiency virus infection, bacterial infection, current smokers, clinical history of peripheral venous thrombosis or Budd-Chiari syndrome, and adherence to fenfluramine or its derivatives, amphetamines, dasatinib, or interferon-alpha.

Diagnosis of pulmonary hypertension and portal hypertension

Pulmonary hypertension was diagnosed as mean pulmonary arterial pressure ≥25 mmHg according to the 2015 European Society of Cardiology and European Respiratory Society (ESC/ERS) Guidelines for the diagnosis and treatment of pulmonary hypertension [4]. Pulmonary hypertension was also measured through echocardiographic measurements as per criteria proposed in the 2015 ESC/ERS Guidelines on pulmonary hypertension. Pulmonary vascular resistance (PVR) was also measured by echocardiography as outlined below. Pulmonary capillary wedge pressure (PCWP) was not measured thus, however mitral E/A ratio was measured and those falling in grade III left ventricular diastolic dysfunction were excluded. Accordingly, patients were divided into those with and without pulmonary hypertension. Portal hypertension was diagnosed according to ultrasound-based criteria. These were: (i) dilated portal vein (>13 mm); (ii) biphasic or reverse flow in portal vein; (iii) portal-systemic collateral pathways (collateral vessels/varices), (iv) splenomegaly; or (v) ascites. Mild/moderate ascites was defined as diuretic responsive whereas severe ascites was defined as diuretic refractory. Echocardiography was performed in the Department of Cardiology by experienced operators with more than five years of experience in echocardiography. It was performed in the left lateral position. The echocardiography machine used was VIVID E95 (GE Healthcare, Chicago, IL, USA).

Laboratory tests

The tests performed were arterial blood gas (ABG), complete blood count (CBC), serum bilirubin, serum albumin, serum glutamic-oxaloacetic transaminase (SGOT), serum glutamic pyruvic transaminase (SGPT), alpha feto protein (AFP), prothrombin time (PT)/ international normalized ratio (INR), serum creatinine, hepatitis B surface antigen (HBsAg), hepatitis C antibody test, and activated partial thromboplastin time (APTT).

Data collection

Data for patient demographics (age, gender, etiology), clinical presentation, laboratory investigations, (blood and serum analysis) and several echocardiographic variables were collected. Echocardiography was performed in line with recommendations outlined by the American Society of Echocardiography in 2015 [7,8]. A comparison of differences in patient demographics, clinical presentation, laboratory findings, and echocardiographic findings was performed in patients with and without portopulmonary hypertension.

Statistical analysis

Continuous variables are presented as mean ± standard deviation, while categorical variables are presented as frequency and percentages. Continuous variables were compared using unpaired t-test. Categorical variables were compared using chi-square test. A p value <0.05 was considered statistically significant. The statistical evaluation of data was done using the using Statistical Package for Social Sciences version 20 (IBM Corp., Armonk, NY, USA).

## Results

Demographic characteristics

Portopulmonary hypertension was prevalent in eight (9.3%) patients among the 86 cirrhotic patients. Portopulmonary hypertension was most prevalent in the 51-60 years age group. There were no gender related differences with regards to prevalence of portopulmonary hypertension. Partial pressure of oxygen (89.12±4.45 mmHg vs. 88.49±5.21 mmHg, p=0.74), carbon dioxide (30.38±6.84 mmHg vs. 33.32±9.73 mmHg, p=0.40) and systolic (100.50±8.19 mmHg vs. 100.23±6.35 mmHg, p=0.91) and diastolic blood pressure (53.50±9.54 mmHg vs. 56.00±7.62 mmHg, p=0.39) did not differ significantly between patients with and without portopulmonary hypertension. Demographic characteristics of the study population are outlined in Table 1.

**Table 1:** Demographic characteristics of the patients with and without portopulmonary hypertension Data are expressed as number (percentage) or mean ± standard deviation PO_2_ - partial pressure of oxygen, PCO_2_ - partial pressure of carbon dioxide

Variable	Portopulmonary Hypertension		p value
	Present (n=8)	Absent (n=78)	
Age			
<40 years	3/33 (9.1%)	30/33 (90.9%)	0.85
40–50 years	1/16 (6.3%)	15/16 (93.8%)	
51–60 years	3/22 (13.6%)	19/22 (86.4%)	
>60 years	1/15 (6.7%)	14/15 (93.3%)	
Male	5/67 (7.5%)	62/67 (92.5%)	0.27
PO_2_,mmHg	89.12±4.45	88.49±5.21	0.74
PCO_2_, mmHg	30.38±6.84	33.32±9.73	0.40
Systolic blood pressure, mmHg	100.50±8.19	100.23±6.35	0.91
Diastolic blood pressure, mmHg	53.50±9.54	56.00±7.62	0.39

Laboratory investigations and etiology

**Figure 1:**
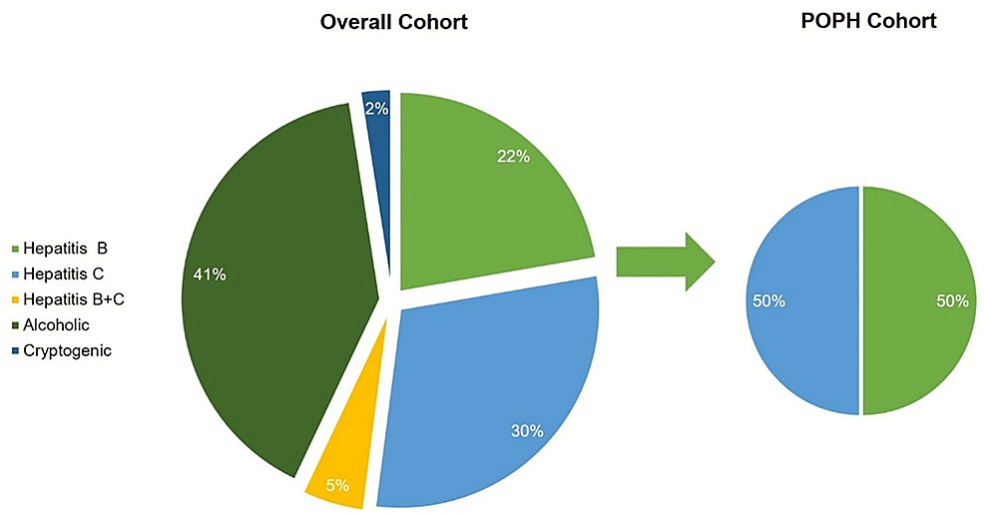
Etiology of cirrhosis among the overall cohort and the portopulmonary cohort POPH - portopulmonary hypertension

Hemoglobin was significantly lower among patients with portopulmonary hypertension compared to those without portopulmonary hypertension (5.50±0.68 g/dl vs. 7.26±1.43 g/dl, p=0.001). Pulmonary hypertension prevalence was observed in two of nine (22.2%) patients with severe ascites which was greater compared to patients with mild or moderate ascites (p=0.20). Hepatitis B was observed in four of eight (50.0%) patients with portopulmonary hypertension and Hepatitic C was observed in four of eight (50.0%) patients with portopulmonary hypertension. No patients with portopulmonary had Class A Child Pugh Turcott severity of cirrhosis, five of eight (62.5%) had Class B Child Pugh Turcott severity of cirrhosis, and three of eight (37.5%) had Class C Child Pugh Turcott severity of cirrhosis. Laboratory investigations are detailed in Table 2. Etiologies of portopulmonary hypertension for the overall cohort and portopulmonary cohort are illustrated in Figure 1.

**Table 2:** Laboratory investigations of patients with and without portopulmonary hypertension Data are expressed as number (percentage) or mean ± standard deviation

Variable	Portopulmonary Hypertension		p value
	Present (n=8)	Absent (n=78)	
Hematological investigations			
Hemoglobin, g/dl	5.50±0.68	7.26±1.43	0.001*
Mean corpuscular volume, fL/cell	73.00±5.85	72.68±4.90	0.86
Mean corpuscular hemoglobin, pg/cell	27.25±2.65	26.96±2.32	0.74
Total leucocyte count, cells/cumm	8558.00±2690.40	9149.62±4604.96	0.72
Platelet count, cells/cumm)	0.84±0.37	0.82±0.34	0.91
Serum glutamic-oxaloacetic transaminase, U/L	196.75±214.92	296.97±192.65	0.21
Serum glutamic-pyruvic transaminase, U/L	191.88±193.02	286.55±193.66	0.19
Serum albumin, g/dL	2.58±0.46	2.51±0.32	0.61
Prothrombin time, sec	21.05±7.42	21.81±7.40	0.78
Serum bilirubin	2.20±0.70	2.34±0.78	0.62
International normalized ratio	1.99±1.04	2.22±1.78	0.72
Child Pugh Turcott Severity			
Class A	0/8 (0%)	0/8 (0%)	0.20
Class B	5/8 (62.5%)	3/8 (37.5%)	
Class C	3/8 (37.5%)	5/8 (62.5%)	
Fibroscan, kPa	50.38±11.37	28.85±10.80	0.0001*

Right atrial and right ventricular dilation and other variables

All patients with portopulmonary hypertension displayed right atrial (major: p=0.0001 and minor: p=0.001) and right ventricular (basal, p=0.0001; longitudinal, p=0.0001) dilation which was statistically significant. Several variables such as right ventricular systolic pressure (p=0.0001), pulmonary artery diameter (major: p=0.0001; right: p=0.0001; and left: p=0.007), pulmonary vascular resistance (p=0.0001), tricuspid regurgitation (p=0.0001), pulmonary regurgitation peak pressure gradient (p=0.0001), pulmonary regurgitation end diastolic gradient (p=0.0001), left atrial dimension (major axis) (p=0.002), left atrial volume (p=0.04), left ventricular outflow tract (p=0.001), inferior vena cava diameter (IVC) (p=0.001), and inferior vena cava collapsibility (p=0.001) were significantly higher in patients with portopulmonary hypertension compared to patients without portopulmonary hypertension. Right atrial and right ventricular dilation and other echocardiographic variables are elaborated in Tables 3 and 4, respectively. The comparison of pulmonary vascular resistance with mean pulmonary arterial pressure is displayed in Figure 3.

**Table 3:** Association right atrial and right ventricular dilation in patients with and without portopulmonary hypertension Data are expressed as number (percentage)

Variable	Portopulmonary Hypertension		p value
	Present (n=8)	Absent (n=78)	
Right atrium dimension major			
Dilated	8/8 (100.0%)	0/8 (0.0%)	0.0001*
Not dilated	0/78 (0.0%)	78/78 (100.0%)	
Right atrium dimension minor			
Dilated	7/7 (100.0%)	0/7 (0.0%)	0.0001*
Not dilated	1/79 (1.3%)	78/79 (98.7%)	
Right ventricle dimension-basal			
Dilated	8/8 (100.0%)	0/8 (0.0%)	0.0001*
Not dilated	0/78 (0.0%)	78/78 (100.0%)	
Right ventricle dimension-mid			
Dilated	8/14 (57.1%)	6/14 (42.9%)	0.0001*
Not dilated	0/72 (0.0%)	72/72 (100.0%)	
Right ventricle dimension-longitudinal			
Dilated	7/7 (100 %)	0/7 (0.0%)	0.0001*
Not dilated	1/79 (1.3%)	78/79 (98.7%)	

**Table 4:** Association right atrial and right ventricular variables in patients with and without portopulmonary hypertension Data are expressed as number (percentage) and mean ± standard deviation

Variable	Present (n=8)	Absent (n=78)	p value
Right ventricular systolic pressure, mmHg	48.92±8.62	33.45±3.39	0.0001*
Pulmonary artery			
Major, cm	2.61±0.12	1.88±0.07	0.0001*
Right, cm	1.85±0.10	1.53±0.13	0.0001*
Left, cm	1.67±0.07	1.55±0.11	0.007*
Pulmonary vascular resistance			
Pulmonary vascular resistance, WU	4.84±1.45	1.05±0.63	0.0001*
Pulmonary vascular resistance, dyn s cm^-5^	387.50±116.26	84.43±50.59	0.0001*
Tricuspid regurgitation, m/sec	3.26±0.31	2.61±0.16	0.0001*
Pulmonary regurgitation peak pressure gradient, mmHg	35.08±7.33	1.96±0.07	0.0001*
Pulmonary regurgitation end diastolic gradient, mmHg	26.93±7.47	4.54±0.11	0.0001*
Left atrial dimension (major axis), cm	2.95±1.11	2.23±0.54	0.002*
Left atrial volume, ml	42.75±8.81	37.99±6.05	0.04*
Left ventricular ejection fraction, %	57.88±2.47	58.21±3.44	0.79
Left ventricular outflow tract, cm	2.22±0.43	1.87±0.20	0.001*
Inferior vena cava diameter	2.35±0.11	1.88±0.20	0.001*
Inferior vena cava collapsibility			
Collapsible	0 (0.0%)	70 (100.0%)	0.001*
Non-collapsible	8 (50.0%)	8 (50.0%)	

**Figure 2:**
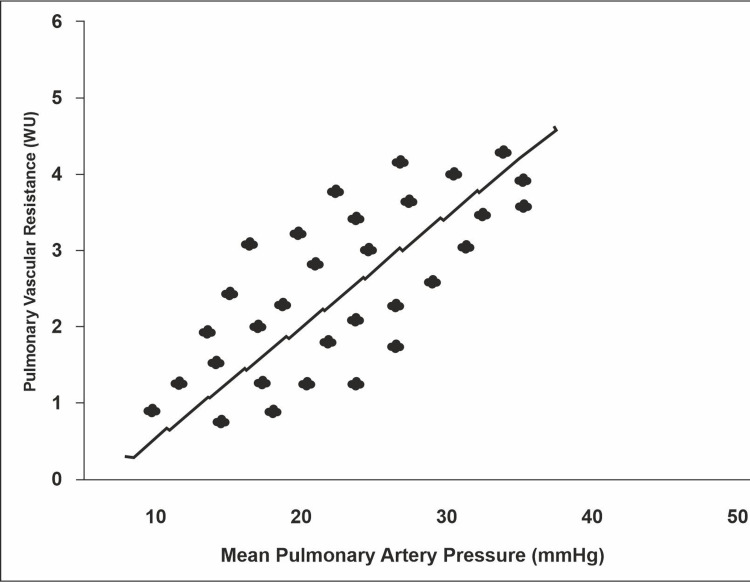
Comparison of pulmonary vascular resistance (Woods units [WU]) with mean pulmonary arterial pressure (mmHg)

## Discussion

The present study explored the prevalence of portopulmonary hypertension among patients with hepatic cirrhosis. The prevalence was found to be 9.3%. Similarly, Chiva et al. [9] reported a 1.7% prevalence in a Spanish population of cirrhotic patients. Shao et al. [10] documented 2.8% prevalence in a Chinese population of cirrhotic patients. Vergara et al. [3] observed a 6.1% prevalence in a Mexican population. Chen et al. [11] observed a 10.0% prevalence in a Chinese population of cirrhotic patients. The prevalence found in the present study is in line with the aforementioned studies. However, disparity among the studies in terms of population, sample size, study design, diagnostic criteria, and diagnostic procedure should be taken into account. The impact of differing yet critical aspects of study design was revealed in a recent study by Atsukawa et al. [2]. Hemodynamic criteria defining pulmonary hypertension have evolved throughout the years. The World Symposium on Pulmonary Hypertension outlined updated hemodynamic criteria to define pulmonary hypertension as increased mean pulmonary arterial pressure ≥20 mmHg (earlier defined as ≥25 mmHg), pulmonary capillary wedge pressure <15 mmHg and pulmonary vascular resistance 240 dyn s cm^5^ [12]. Atsukawa et al. [2] reported a 1.1% and 3.3% prevalence of portopulmonary hypertension in a Japanese population of cirrhotic patients according to earlier and later criteria. This evidences that dissimilarities among studies may either underestimate or overestimate the prevalence of portopulmonary hypertension. The clinical implications of the newer criteria in a real-world clinical scenario still warrant further investigation.

The prevalence of portopulmonary hypertension was higher among females than males, although not statistically significant. It is possible our study lacked statistical significance owing to the small sample size. However, this finding was found to be statistically significant in the study by Shao et al. [10]. Moreover, Vergara et al. [12] and Kawut et al. [13] revealed female gender to be associated with a higher risk of portopulmonary hypertension than male gender. Earlier studies have attributed this higher prevalence to the role of estrogen in the disease [14].

Hepatitis B was the most common etiology. This observation is further fortified by other studies [10,11]. Hepatitis C was the most common cause reported by Chiva et al. [9]. In contrast, hepatitis C has been postulated to play a protective role in the risk of portopulmonary hypertension [13].

Left ventricular dilatation can be explained by higher severity of anemia seen in portopulmonary hypertension patients [15]. In line with this observation in literature, hemoglobin was statistically lower in patients with portopulmonary hypertension. The same observation was revealed in the study by Shao et al. [10]. This observation was further fortified by Chen et al. [11] who also identified hemoglobin as an independent risk factor.

A numerical increase in Child-Pugh scoring classification was observed in the present study, which was found to be statistically significant. This infers that portopulmonary hypertension was observed in all Child-Pugh classes. Although one study has shown a weak association between portopulmonary hypertension and the severity of liver disease [16,17], several other studies reported the risk of portopulmonary hypertension did not increase with the aggravation of the Child-Pugh classification [2,9-11,18]. A common observation of several studies was majority presence of patients in Class B [3,5,6,12,14].

Study limitations

The present study is limited by the small sample size. In the time spanning the conduction of this study and its publication, the latest guidelines redefining portopulmonary hypertension have been published. The impact of these guidelines in clinical practice warrant further investigations in a population of greater sample size. Secondly, computed tomography (CT) thorax was not performed to rule out interstitial lung disease or ` which can also independently cause pulmonary hypertension. Lastly, right heart catheterization remains the gold standard for the diagnosis of pulmonary hypertension. It is an invasive as well as resource intensive method. However, cardiac catheterization-derived PVR index (PVRI)/PA mean are more representative. These drawbacks have led to echocardiography as a more preferred method. This imaging modality has further been supported by recent literature [19].

## Conclusions

The present study revealed a 9.3% prevalence of portopulmonary hypertension among patients with liver cirrhosis. Hemoglobin was significantly lower among patients with portopulmonary hypertension compared to those without portopulmonary hypertension. All patients with portopulmonary hypertension displayed right atrial and right ventricular dilation. Moreover, several echocardiographic variables were significantly higher in patients with portopulmonary hypertension compared to patients without portopulmonary hypertension.
